# Low baseline ischemic water uptake is directly related to overestimation of CT perfusion-derived ischemic core volume

**DOI:** 10.1038/s41598-022-19176-7

**Published:** 2022-11-29

**Authors:** Rosalie McDonough, Sarah Elsayed, Lukas Meyer, Theresa Ewers, Matthias Bechstein, Helge Kniep, Marie Teresa Nawka, Tobias D. Faizy, Gerhard Schön, Götz Thomalla, Jens Fiehler, Uta Hanning, Andre Kemmling, Gabriel Broocks

**Affiliations:** 1 Department of Diagnostic and Interventional Neuroradiology, University Medical Center Hamburg-Eppendorf, Martinistrasse 52, 20246 Hamburg, Germany; 2 Institute of Medical Biometry and Epidemiology, University Medical Center Hamburg-Eppendorf, Hamburg, Germany; 3 Department of Neurology, University Medical Center Hamburg-Eppendorf, Hamburg, Germany; 4 Department of Neuroradiology, Westpfalz-Klinikum, Kaiserslautern, Germany

**Keywords:** Cell death in the nervous system, Diseases of the nervous system, Cerebrovascular disorders, Stroke

## Abstract

Computed-tomography perfusion (CTP) is frequently used to screen acute ischemic stroke (AIS) patients for endovascular treatment (EVT), despite known problems with ischemic “core” overestimation. This potentially leads to the unfair exclusion of patients from EVT. We propose that net water uptake (NWU) can be used in addition to CTP to more accurately assess the extent and/or stage of tissue infarction. Patients treated for AIS between 06/2015 and 07/2020 were retrospectively analyzed. Baseline CTP-derived core volume (pCore) and NWU were determined. Logistic regression tested the relationship between baseline clinical and imaging variables and core-overestimation (primary outcome). The secondary outcomes comprised 90-day functional independence (modified Rankin score) and lesion growth. 284 patients were included. Median NWU was 7.2% (IQR 2.6–12.8). ASPECTS (RR 1.28, 95% CI 1.09–1.51), NWU (RR 0.94, 95% CI 0.89–0.98), onset to recanalization (RR 1.00, 95% CI 0.99–1.00) and imaging (RR 1.00, 95% CI 1.00–1.00) times, and pCore (RR 1.02, 95% CI 1.01–1.02) were significantly associated with core overestimation. Core-overestimation was more likely to occur in patients with large pCores and low NWU at baseline. NWU was significantly correlated with lesion growth. We conclude that NWU can be used as a supplemental tool to CTP during admission imaging to more accurately assess the extent of ischemia, particularly relevant for patients with large CTP-defined cores who would otherwise be excluded from treatment.

## Introduction

Advanced imaging is often used for the treatment selection of patients with acute ischemic stroke (AIS) due to large vessel occlusion (LVO) by identifying the volume of ischemic yet potentially salvageable tissue (penumbra). Despite recent breakthroughs in AIS therapy regimes with the extension of the acceptable time window for endovascular treatment (EVT), the dynamics of the infarct growth curve remain unknown^1–3^. It has been proposed that some patients are “fast progressors”, presenting with large volume infarctions within a relatively narrow time window, while others retain tissue viability despite long vessel occlusion times (i.e., “slow progressors”)^4^. In almost all cases, however, the vast majority will undergo irreversible tissue damage if left untreated^4,5^.

Certain computed tomography perfusion (CTP)-based parameters, such as cerebral blood volume (CBV) or cerebral blood flow (CBF) are frequently employed to assess the extent of irreversibly damaged tissue during admission imaging, particularly for those presenting within the extended time window (> 6 h)^6^. It is thought that the larger the ischemic lesion, as well as the smaller the penumbral mismatch, the riskier it is to perform an invasive procedure. Indeed, in the DAWN and DEFUSE III landmark trials, patients were stratified for treatment selection based on the CT or MR-derived size of the infarct core, the ratio of core to penumbra, and/or the mismatch between clinical severity and imaging results^2,3^. As a result, patients with large cores (e.g., potential “fast progressors”), as well as those with minimal clinical deficits (potential “slow progressors”) were excluded from these studies. In fact, relatively little is known about the benefit of treatment in such patient groups.

Despite the consequences for individual patients, the question of validity of such methods remains^7^. In addition to providing limited spatial resolution, CBV and CBF have been repeatedly observed to overestimate final infarct size (“ghost infarct core” phenomenon), particularly within the early time window^8–10^. Net water uptake (NWU) has recently emerged as an attractive quantitative imaging biomarker^11,12^. Not only is it easily and quickly assessed, but it is based on pathophysiological processes leading to increased vasogenic edema and, as a result, is specific for irreversible tissue injury. When taken together, NWU and CTP-based core estimation parameters could provide a more accurate picture of both lesion size and stage of infarction at the admission timepoint. We hypothesize that low levels of NWU are predictive of overestimation within the CTP-defined core lesion.

## Methods

### Study population

This represents a single-center retrospective observational cohort study of patients suffering from AIS due to LVO, consecutively treated at the University Medical Center Hamburg-Eppendorf between June 2015 and July 2020. Patient selection was done based on the following a priori defined inclusion criteria: (1) age > 18 years, (2) multimodal CT (non-contrast CT (NCCT), CT angiography (CTA), and CTP) performed at admission; (3) absence of intracranial hemorrhage (4) endovascular treatment with or without prior intravenous (i.v.) recombinant tissue plasminogen activator (rtPA); and (5) follow-up imaging (NCCT) performed within 24 h of admission. Patient baseline characteristics were extracted from the medical records and clinical outcome parameters (modified Rankin scale (mRS) at discharge and 90 days, mRS90) were documented, if available. The local ethics review board of the University Medical Center Hamburg-Eppendorf approved the use of anonymized patient data for this retrospective analysis and waived the requirement for informed consent. All study protocols and procedures were conducted in accordance with ethical guidelines of the University Medical Center Hamburg-Eppendorf and in compliance with the Declaration of Helsinki.

### Image acquisition

All patients received multimodal stroke imaging at admission with NCCT, CTA, and CTP performed in equal order on 256 or 384 dual slice scanners (Philips iCT 256, Siemens Somatom Force). NCCT: 120 kV, 280 to 340 mA, 5.0 mm slice reconstruction, 1-mm increment; CTA: 100 kV, 260 to 300 mA, 5.0 mm slice reconstruction, 1-mm increment, 80 mL highly iodinated contrast medium and 50 mL NaCl flush at 4 mL/s; CTP: 80 kV, 200 to 250 mA, 5 mm slice reconstruction (maximum 10 mm), slice sampling rate 1.50 s (minimum 1.33 s), scan time 45 s (maximum 60 s), biphasic injection with 30 mL (maximum 40 mL) of highly iodinated contrast medium with 350 mg iodine/mL (maximum 400 mg/mL) injected with at least 4 mL/s (maximum 6 mL/s) followed by 30 mL sodium chloride chaser bolus, whole-brain coverage of 12 cm. All perfusion datasets underwent quality control and were excluded in case of severe motion artifacts.

### Image analysis

Anonymized CT imaging data was segmented using semiautomatic commercially available software (Analyze 11.0, Biomedical Imaging Resource, Mayo Clinic, Rochester, MN). The raters (RM, SE) were blinded for all other imaging data and patient information. CTP-guided delineation of ischemic lesion NWU on admission imaging was determined according to recently published methods^11,13^. In brief, the visually evident, circumscribable hypoattenuated ischemic core lesion on NCCT was assessed by densitometric measurements (D_ischemic_). A corresponding region of interest was mirrored symmetrically on the contralateral, non-affected hemisphere (D_normal_). In cases where early hypoattenuation was not visibly evident, the CTP-generated CBV map was used as a visual guide for the selection of the region of interest (ROI). ROI histograms were sampled between 20 and 80 Hounsfield units (HU) to exclude voxels that correspond to cerebrospinal fluid or calcifications. The density measurements, D_infarct_ and D_normal_, were then used to calculate percent NWU (%NWU, Eq. 1), i.e., the proportion of water uptake within the infarct lesion compared to the contralateral side.

1 $$\% {\text{NWU}} = \left( {1{-}{\text{D}}_{{{\text{ischemic}}}} /{\text{D}}_{{{\text{normal}}}} } \right) \times 100\%$$

Raw perfusion data were analyzed on a Siemens^®^ workstation using Syngo^®^ VPCT *Neuro* software (Siemens Healthcare, Erlangen, Germany). Quantitative maps of relative CBF (rCBF), CBV, MTT, and Tmax were generated using a delay-insensitive algorithm. For comparability, we selected a predicted core (pCore) threshold of rCBF ≤ 20%, as this has been described to have the highest level of agreement with RAPID-generated rCBF-based volumes^14^. Hypoperfusion volume was determined to be the volume of tissue with a prolonged Tmax of at least 6 s (Tmax > 6 s). Mismatch (penumbral) volume was defined as the difference between this volume and pCore.

The final infarct lesion volume (FIV) on follow-up imaging (NCCT) was determined by manual segmentation using semiautomatic commercially available software (Analyze 11.0, Biomedical Imaging Resource, Mayo Clinic, Rochester, MN). Finally, lesion growth between admission and follow-up imaging was determined by calculating the difference between the FIV and pCore (Eq. 2).

2 $${\text{Lesion}}\;{\text{growth}} = {\text{FIV}}{-}{\text{pCore}}$$

### Statistical analysis and outcome measures

Descriptive analyses were used to define population baseline, imaging, and procedural characteristics. Shapiro–Wilk and histograms tested for the normality of distributions. The primary outcome measure was the binarized core overestimation variable; if lesion growth was negative by more than 10 ml (chosen in accordance with a previous study^9^ and to account for segmentation error), the pCore was scored as being overestimated. The secondary outcomes were good functional outcome at 90 days, mRS90 0–2, and lesion growth. Because the volumetric measurements of lesion growth were heavily skewed, the cubic root was taken for the analysis. Linear (cubic root of lesion growth) and binary logistic regression models (core overestimation and favorable outcome, mRS90 0–2) were used to test the interactions. Significant predictors of outcome variables in univariable regression were used to construct the multivariable models. A sensitivity analysis was performed following stratification of the cohort according to pCore volume ≥ 50 mL versus < 50 mL. This cut off was chosen to be in line with the inclusion criteria of multiple prospective clinical trials, as well as observations from the HERMES metaanalysis^2,15,16^.

All analyses were performed using Stata 15.1 (StataCorp LLC). Normally distributed variables are displayed as mean and standard deviation (SD). Non-normally distributed data are displayed as median and interquartile range (IQR). Categorical variables are reported as proportions. Binary logistic regression results are presented as relative risks (RR) with 95% confidence intervals (95%CI). *p*-values < 0.05 were considered significant.

## Results

### Patient characteristics

284 patients fulfilled the inclusion criteria (for inclusion flowchart, please see Supplementary Figure S1). The median age was 76.5 years (IQR 65–82) and 48% (135) were female. The median pCore was 11.9 mL (IQR 3.6–31.1), measured after a median time from onset to imaging of 3.2 h (IQR 1.3–4.7). A median NWU of 7.2% (IQR 2.6–12.8) was observed and the cohort had a median lesion growth of 18.4 ml (IQR − 1.8–118.8). 49.3% (138/280) underwent successful reperfusion, defined as an expanded Thrombolysis in Cerebral Infarction (eTICI) score of 2c/3. Median FIV was 32.8 mL (IQR 5.7–146.2). Good outcome was observed in 29.3%. Patients with pCore overestimation had significantly higher ASPECTS (8 vs. 7), lower NWU (5.6% vs. 7.6%), larger pCores (34.2 mL vs. 8.7 mL), and lower rates of successful reperfusion (39.5% vs. 51%) (Table 1). For baseline characteristics of the entire cohort and stratified according to pCore volume, see Supplementary Tables S1 and S2.

**Table 1:** Baseline, imaging, and procedural characteristics of the study sample (n = 284), stratified by core overestimation.

Variable*	Core overestimation–yes (n = 45)	Core overestimation–no (n = 229)
Age (years)–median (IQR)	78 (67–82)	76 (64–82)
mRS before admission–median (IQR)	0 (0–2), n = 39	0 (0–1), n = 189
NIHSS–median (IQR)	16 (15–29)	15 (11–19), n = 220
Systolic blood pressure (mmHg)–mean (SD)	158 (35)	161 (28), n = 219
Diastolic blood pressure (mmHg)–mean (SD)	86 (18)	86 (15), n = 217
Weight (Kg)–median (IQR)^†^	70 (62–80), n = 38	80 (70–89), n = 164
ASPECTS–median (IQR)^†^	8 (7–9)	7 (6–9), n = 228
NWU (%)–median (IQR)^†^	5.6 (1.3–7.8)	7.6 (2.8–13.7)
pCore (mL)–median (IQR)^†^	34.2 (25.0–67.2)	8.7 (2.3–25.6)
Penumbra (mL)–median (IQR)	83.9 (49.1–141.1)	70.6 (43.6–105.9)
Successful reperfusion (eTICI 2c/3)–n (%)^†^	17 (39.5%)	121 (51%)
mRS90 0–2–n (%)	16 (42.1%), n = 38	47 (27.0%), n = 174

*IQR* interquartile range; *mRS* modified Rankin scale; *NIHSS* National Institutes of Health Stroke Scale; *ASPECTS* Alberta Stroke Program Early CT Score; *NWU* net water uptake; *pCore* CTP-defined core volume, *eTICI* expanded Thrombolysis in Cerebral Infarction scale.

* n is provided only in case of missing values.

^†^Wilcoxon rank sum test *p* < 0.05.

### Association of variables with pCore overestimation

**Figure 1:**
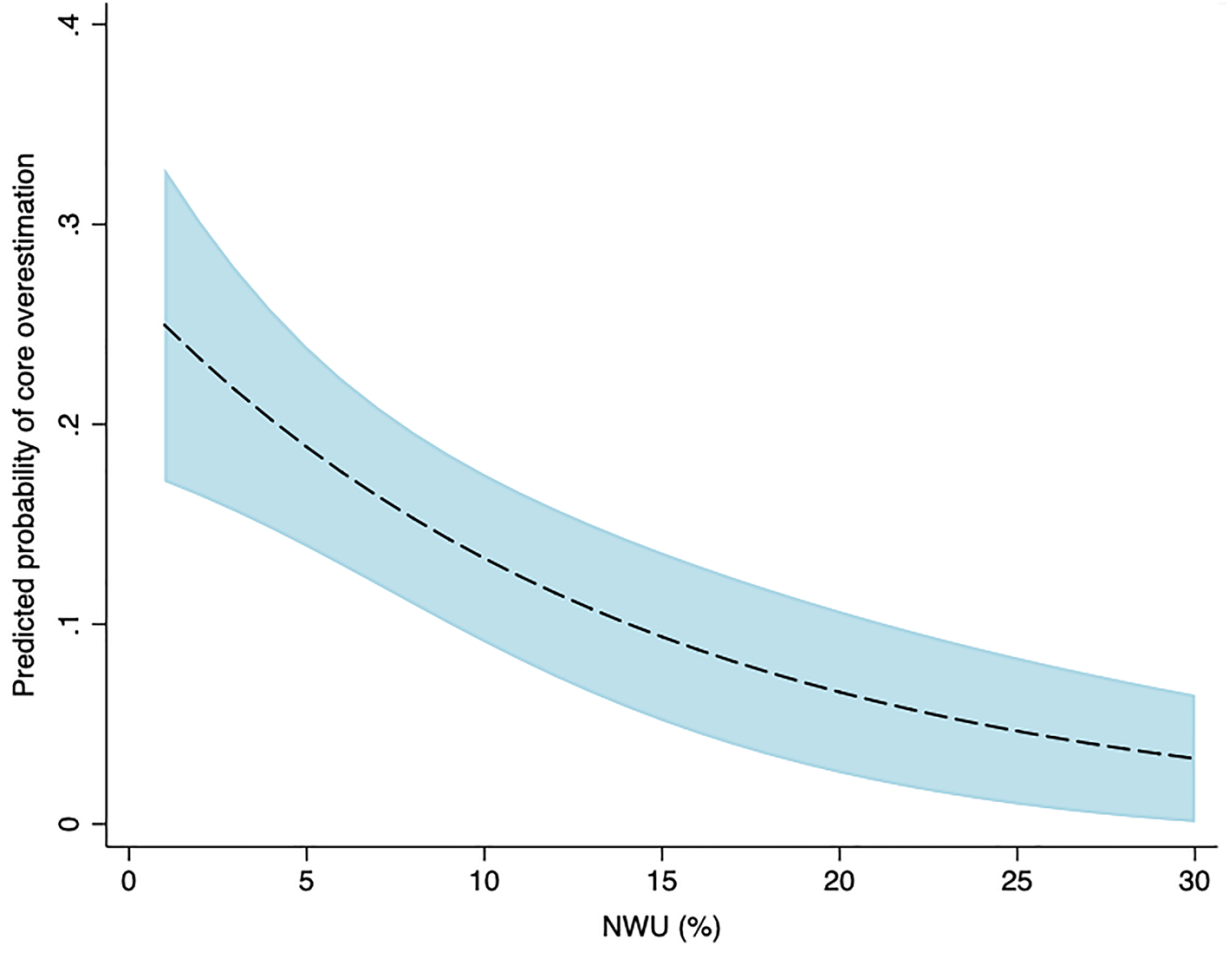
Predicted probability of core overestimation by NWU at admission. Lower levels of NWU were significantly and positively associated with core overestimation. *NWU* net water uptake*.*

In univariable logistic regression, ASPECTS (RR 1.21, 95% CI 1.05–1.39), pCore (RR 1.01, 95% CI 1.01–1.02), NWU (RR 0.93, 95% CI 0.90–0.97), onset to imaging (RR 1.00, 95% CI 0.99–1.00), and onset to recanalization (RR 0.99, 95% CI 0.99–1.00) were significantly associated with core overestimation (Fig. 1, Table 2). These associations remained significant in multivariable regression modelling (Table 2).

**Table 2:** Univariable and multivariable binary logistic regression analysis of the effect of baseline, imaging, and procedural variables on pCore overestimation and good functional outcome (mRS 0–2).

Predictor	Core overestimation		Good outcome (mRS 0–2) at 90 days	
	Unadjusted RR (95% CI)	Adjusted RR* (95% CI)	Unadjusted RR (95% CI)	Adjusted RR* (95% CI)
Age (years)	1.02 (0.99–1.04)	–	**0.97 (0.96–0.98)**	**0.97 (0.96–0.98)**
Sex	0.79 (0.46–1.35)	–	1.27 (0.83–1.94)	**–**
NIHSS	1.02 (0.99–1.05)	–	**0.93 (0.90–0.96)**	**0.95 (0.92–0.99)**
ASPECTS	**1.21 (1.05–1.39)**	**1.28 (1.09–1.51)**	**1.2 (1.11–1.37)**	**1.18 (1.06–1.31)**
pCore	**1.01 (1.01–1.02)**	**1.02 (1.01–1.02)**	0.99 (0.98–1.00)	–
NWU (%)	**0.93 (0.90–0.97)**	**0.94 (0.89–0.98)**	0.98 (0.95–1.01)	–
Intravenous alteplase	1.23 (0.76–1.96)	–	1.55 (0.98–2.45)	–
Onset to imaging (min)	**1.00 (0.99–1.00)**^**§**^	**1.00 (1.00–1.00)**^**§**^	1.00 (1.00–1.00)	–
Onset to recanalization (min)	**1.00 (0.99–1.00)**^**§**^	**1.00 (0.99–1.00)**^**§**^	1.00 (1.00–1.00)	–
Successful reperfusion (eTICI 2c/3)	1.18 (0.69–2.01)	–	**1.61 (1.05–2.46)**	**1.55 (1.06–2.28)**

*mRS* modified Rankin Scale; *RR* relative risk; *NIHSS* National Institutes of Health Stroke Scale; *ASPECTS* Alberta Stroke Program Early CT Score; *NWU* net water uptake; *eTICI* expanded Thrombolysis in Cerebral Infarction.

*Significant predictors in univariable regression were used to build the multivariable model.

^**§**^Values are rounded to the nearest decimal.

Significant associations are shown in bold.

**Figure 2:**
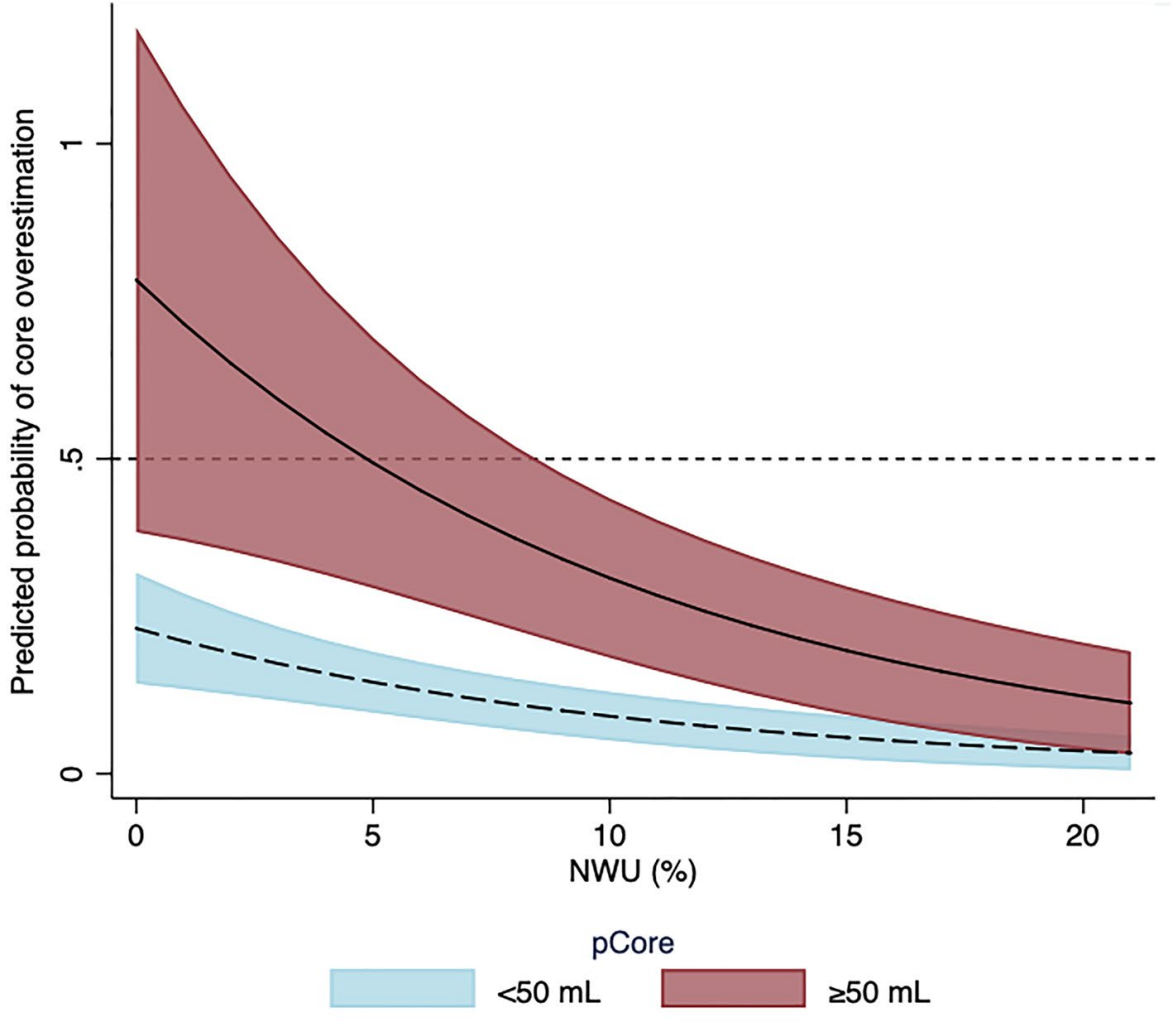
Predicted probability of core overestimation by NWU at admission, stratified by pCore volume. Lower levels of NWU were significantly and positively associated with core overestimation, particularly for those patients with a large pCore ≥ 50 mL. *NWU* net water uptake*.*

**Figure 3:**
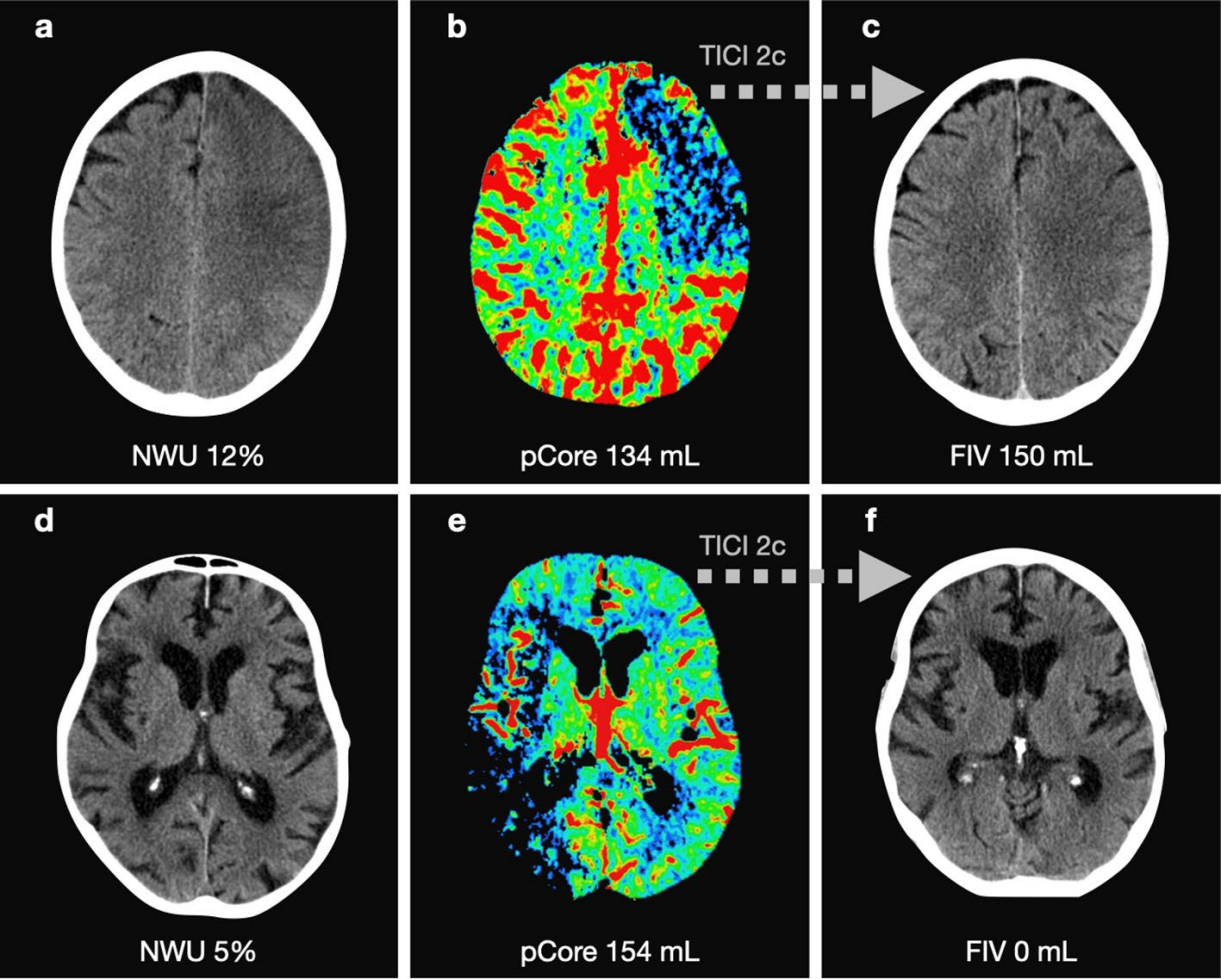
Examples of patients with high (**A**–**C**) and low (**D**–**F**) NWU and their association with pCore overestimation. Both patients presented within a similar time frame and achieved near-completed reperfusion. (**A**, **D**: admission imaging; **B**, **E**: rCBF < 20% CTP maps; **C**, **F**: follow-up imaging). *NWU* net water uptake; *pCore* rCBF20%-defined core at admission; *CTP* CT perfusion.

Following stratification, an association between age, ASPECTS, eTICI grade, pCore, and NWU and core overestimation was observed in the subgroup of patients with a pCore ≥ 50 mL, while NIHSS, pCore, and NWU were the only significant predictors of core overestimation in the small core (pCore < 50 mL) subgroup (Figs. 2 and 3, Supplementary Table S3).

### Association of variables with favorable outcome, mRS 0–2 at 90 days

Univariable logistic regression revealed age (RR 0.97, 95% CI 0.96–0.98), NIHSS (RR 0.93, 95% CI 0.90–0.96), ASPECTS (RR 1.2, 95% CI 1.11–1.37), and successful reperfusion (RR 1.61, 95% CI 1.05–2.46) to be significantly associated with favorable outcome at 90 days; these associations remained stable in a multivariable logistic regression model including all the above parameters and following adjustment for sex (Table 2).

In the small core subgroup (pCore < 50 mL), there were no differences in the variables that were observed to be associated with an mRS90 of 0–2 for the overall patient sample. For those with a pCore ≥ 50 mL, however, only age was a significant predictor of favorable outcome (Supplementary Table S4).

### Association of variables with lesion growth

**Figure 4:**
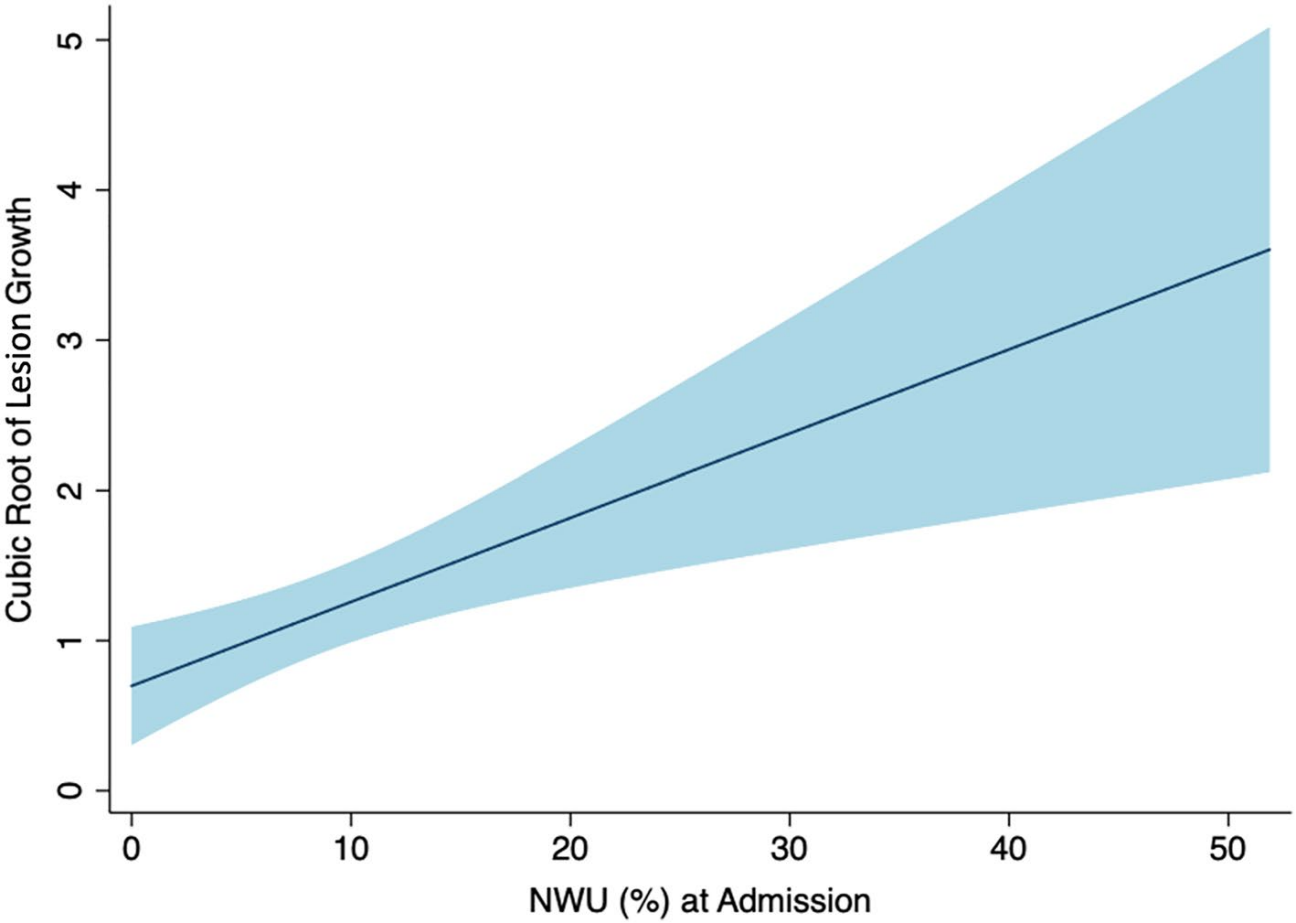
Relationship between NWU and lesion growth. Lower NWU was significantly associated with less lesion growth. *NWU* net water uptake.

Significant associations were observed for ASPECTS, successful recanalization, NWU, pCore, and the cubic root of lesion growth (Table 3). Linear regression analysis showed that increasing NWU led to an increase in the cubic root of lesion growth (ß 0.06, 95% CI 0.02–0.09, *p* = 0.001, Fig. 4, Table 3). The sensitivity analysis showed that ASPECTS, NWU, and successful reperfusion were significantly associated with lesion growth in both the large and small core subgroups (Supplementary Table S5).

**Table 3:** Univariable and multivariable linear regression analysis of baseline, imaging, and procedural variables with the cubic root of lesion growth.

Variable	ß-coefficient (95% CI)	Adjusted ß-coefficient* (95% CI)
Age (years)	**− **0.01 (**− **0.04–0.01)	–
Sex	0.39 (**− **0.15–0.92)	–
NIHSS	**− **0.001 (**− **0.04–0.04)	–
ASPECTS	**− 0.33 (− 0.46**–**[− 0.20])**	**− 0.43 (− 0.57**–**[− 0.30])**
NWU (%)	**0.06 (0.02–0.09)**	**0.04 (0.01–0.07)**
pCore	**− 0.01 (− 0.02**–**[− 0.002])**	**− 0.02 (− 0.03**–**[− 0.01])**
Time onset to imaging (min)	0.001 (**− **0.0003–0.002)	–
Time onset to recanalization (mins)	0.001 (**− **0.001–0.002)	–
Successful reperfusion (eTICI 2c/3)	**− 0.87 (− 1.39**–**[− 0.34])**	**− 0.86 (–1.33**–**[− 0.38])**

*NIHSS* National Institutes of Health Stroke Scale; *ASPECTS* Alberta Stroke Program Early CT Score; *NWU* net water uptake; *eTICI* expanded Thrombolysis in Cerebral Infarction.

*Significant predictors in univariable regression were used to build the multivariable model.

Significant associations are shown in bold.

## Discussion

The purpose of this study was to investigate the relationship between NWU on admission imaging and the probability of CTP-based ischemic core overestimation. We hypothesized that patients with large cores, yet low NWU were the most likely to show core overestimation. The main findings of this study are shown Fig. 2; in this patient cohort, low NWU was indeed significantly associated with core overestimation, thus supporting our hypothesis. Interestingly, this relationship appeared to be particularly robust for the subgroup of patients with large baseline ischemic cores. We also observed that patients with higher levels of NWU on admission imaging had increased lesion growth, although this relationship was no longer significant following adjustment for patient baseline characteristics and reperfusion status. Despite this, these data support a more granular depiction of lesion growth dynamics and propose a multi-stage chronological approach to ischemic core lesion imaging, based on known metabolic processes. Initially, the cells distal to the arterial occlusion undergo oxygen deprivation, resulting in a compensatory increase in CBF. Over time, however, these compensatory mechanisms fail, leading to a progressive decline in CBF and CBV, with transformation of the penumbra into irreversibly damaged tissue. Due to continued cellular oxygen deprivation, disruption of the blood brain barrier ensues, leading to the influx of ions and subsequently tissue water uptake. The combined assessment of CTP parameters and NWU could reflect these stages; in patients with large CBV deficits, but low NWU, the compensatory mechanisms are largely maintained–the core lesion is “reversible”. On the other hand, those with large CBV lesions and high NWU have more likely crossed the threshold into irreversible tissue damage. It is possible that these patients also experience more growth due to a developing increase of NWU within “borderline” penumbral tissue, signaling initial breakdown of the blood–brain-barrier, which was not assessed in this study. Furthermore, the current definition of core volume is based on binary thresholding, thereby likely oversimplifying complex pathophysiological processes^17^. It would be interesting, therefore, to examine whether there is a “gradient” of NWU around the CBV-derived core lesion.

The associations of age, NIHSS, ASPECTS, and successful reperfusion with favorable outcome confirm that which has been extensively shown in the literature^18–20^. While NWU was not a significant predictor of mRS90 0–2 in this patient cohort, its potential role in the improved selection of patients most likely to benefit from EVT would be of substantial value, particularly for those who would have otherwise been excluded due to the results of advanced imaging. Indeed, ASPECTS represents a binary measure of early ischemic changes, not taking the degree of hypodensity into account. Performing concomitant NWU analyses could provide a more detailed overview of the extent of tissue damage at the time of baseline imaging.

Patients with large areas of severe hypoattenuation (i.e., high water uptake) are frequently excluded from EVT, and thus underrepresented in this study. Nevertheless, in a sensitivity analysis restricted to the patient subgroup with a pCore ≥ 50 mL, successful reperfusion was found to be negatively associated with lesion growth, with younger patients more likely to achieve a good outcome. This points toward a potential benefit of EVT, even for those with large baseline infarctions. The currently running randomized trials, including TESLA (ClinicalTrials.gov Identifier: NCT03805308), TENSION (ClinicalTrials.gov Identifier: NCT03094715), IN-EXTREMIS-LASTE (ClinicalTrials.gov Identifier: NCT03811769), and SELECT-2 (ClinicalTrials.gov Identifier: NCT03876457) will hopefully soon provide evidence for such cases.

This study has limitations. First, despite the long recruitment period, relatively few patients were included. This was particularly true for the large core subgroup, as such patients rarely undergo EVT. This likely resulted in the sensitivity analysis being underpowered to detect potential predictors of outcome and lesion growth. Nevertheless, we believe the significant association between NWU and core overestimation to be of particular interest for this patient population. Second, our standard acute stroke protocol consists of NCCT, CTA, and CTP independent of time window (i.e., is also done for patients presenting within 6 h). This should lend caution to stroke physicians in their decision-making processes, as the use of advanced imaging in the early time window could result in false exclusion of patients. Third, the association between patient clinical, imaging, and procedural characteristics with lesion growth may have been confounded by the potentially inaccurate measurements of pCore. To this end, we adjusted the analyses for the initial core measurements. Fourth, we included patients with both successful (eTICI 2c/3) and unsuccessful reperfusion in our cohort, which likely impacted infarct growth dynamics. Fourth, due to the retrospective nature of the study and relatively long recruitment period, there were substantial missing data, particularly for the 3-month clinical outcome, mRS90.

To our knowledge, this is the first study that examines the modifying effect of NWU on CTP-derived core overestimation in patients with AIS who underwent EVT. This relationship of NWU on infarct core lesion size and growth is particularly relevant for those subsets of patients that have been, if inadvertently, excluded from the recent large clinical trials. If CTP-based parameters are to remain a major part of the decision-making workflow in AIS, a certain level of accuracy must be achieved. The relatively easy adjunct calculation of NWU could help clinicians decide which patients would most likely benefit from EVT thereby further contributing to the personalization of stroke medicine.

## Conclusion

NWU can be used as a supplemental tool to CTP during admission imaging to more accurately assess the extent of irreversible ischemia, particularly relevant for patients with large CTP-defined cores who would otherwise be excluded from treatment.

## Supplementary Information

Supplementary Information.

## Data Availability

Data are available from the corresponding author upon reasonable request.
