# Supplementary material for: Low baseline ischemic water uptake is directly related to overestimation of CT perfusion-derived ischemic core volume
Source: Sci Rep. 2022 Nov 29;12:20567. doi: 10.1038/s41598-022-19176-7 (PMC9708677; doi:10.1038/s41598-022-19176-7)
Supplement: Supplementary file 1 — Supplementary Information. [file 41598_2022_19176_MOESM1_ESM.pdf]

Low baseline ischemic water uptake is directly related to overestimation of CT perfusion-derived ischemic core volume

Supplementary Material

Supplementary Table S1: Baseline characteristics of the study sample (n = 284).

**Variable***	
Age (years) – median (IQR)	76.5 (65 – 82)
mRS before admission – median (IQR)	0 (0 – 1), n = 230
NIHSS – median (IQR)	16 (11 – 19), n = 275
Systolic blood pressure (mmHg) – mean (SD)	160 (29), n = 274
Diastolic blood pressure (mmHg) – mean (SD)	86 (15), n = 272
Weight (Kg) – median (IQR)	79 (68 – 86), n = 210
ASPECTS – median (IQR)	8 (6 – 9), n = 281
NWU (%) – median (IQR)	7.2 (2.6 – 12.8)
pCore (mL) – median (IQR)	11.9 (3.6 – 31.1)
Penumbra (mL) – median (IQR)	70.5 (44.2 – 112.3)
Successful reperfusion (eTICI 2c/3) – n (%)	138 (49.3%), n = 280
mRS90 0-2 – n (%)	63 (29.3%), n = 215

* n is provided only in case of missing values

IQR: interquartile range; mRS: modified Rankin scale; NIHSS: National Institutes of Health Stroke Scale; ASPECTS: Alberta Stroke Program Early CT Score; NWU: net water uptake; pCore: CTP-defined core volume, eTICI: expanded Thrombolysis in Cerebral Infarction scale

Supplementary Table S2. Baseline characteristics of the study sample, stratified according to pCore volume.

Variable*	Core ≥50 mL, n = 43	Core <50 mL, n = 241
Age (years) – median (IQR)	73 (62 – 80)	77 (65 – 83)
mRS before admission – median (IQR)	0 (0 – 1), n = 37	0 (0 – 1), n = 193
NIHSS – median (IQR)**	18 (15 – 21)	15 (10 – 19), n = 232
Systolic blood pressure (mmHg) – mean (SD)	160 (28)	160 (29), n = 231
Diastolic blood pressure (mmHg) – mean (SD)	85 (14)	86 (15), n = 229
Weight (Kg) – median (IQR)	80 (70 – 89), n = 31	79 (68 – 86), n = 179
ASPECTS – median (IQR)**	6 (5 – 7)	8 (7 – 9), n = 239
NWU (%) – median (IQR)**	9.4 (7.2 – 12.0)	6.2 (2.0 – 13.2)
pCore (mL) – median (IQR)**	72.0 (59.5 – 103.8)	8.1 (2.1 – 19.3)
Penumbra (mL) – median (IQR)	83.9 (57.1 – 140.8)	68.8 (43.6 – 109.3)
Successful reperfusion (eTICI 2c/3) – n (%)	17 (39.5%)	121 (51%)
mRS90 0-2 – n (%)	5 (15.2%), n = 33	58 (31.9%), n =182

* n is provided only in case of missing values

** Wilcoxon rank sum test $p < 0.05$

IQR: interquartile range; mRS: modified Rankin scale; NIHSS: National Institutes of Health Stroke Scale; ASPECTS: Alberta Stroke Program Early CT Score; NWU: net water uptake; pCore: CTP-defined core volume, eTICI: expanded Thrombolysis in Cerebral Infarction scale

Supplementary Table S3. Associations between variables and binarized core overestimation, stratified by pCore volume.

Variable	Relative Risk (95% CI)	
	Core ≥50 mL	Core <50 mL
Age (years)	**1.04 (1.00-1.07)**	1.01 (0.98-1.04)
NIHSS	0.94 (0.89-1.00)	**1.04 (1.01-1.08)**

ASPECTS	**1.6 (1.33-1.93)**	1.21 (0.99-1.46)
NWU (%)	**0.90 (0.84-0.97)**	**0.92 (0.87-0.97)**
eTICI grade	**1.44 (1.10-1.89)**	1.13 (0.93-1.38)
Successful reperfusion (eTICI 2c/3)	1.75 (0.77-3.96)	1.09 (0.56-2.14)

Significant associations are shown **in bold.**

NIHSS: National Institutes of Health Stroke Scale; ASPECTS: Alberta Stroke Program Early CT Score; NWU: net water uptake; eTICI: expanded Thrombolysis in Cerebral Infarction scale

Supplementary Table S4. Associations between variables and binarized good outcome (mRS90 0-2), stratified by pCore volume.

**Variable**	**Relative Risk (95% CI)**	
	**Core ≥50 mL**	**Core <50 mL**
Age (years)	**0.96 (0.92-0.99)**	**0.97 (0.96-0.98)**
NIHSS	1.07 (1.00-1.14)	**0.92 (0.90-0.95)**
ASPECTS	1.07 (0.78-1.45)	**1.24 (1.10-1.39)**
NWU (%)	1.04 (0.95-1.12)	0.98 (0.95-1.01)
Successful reperfusion (eTICI 2c/3)	0.90 (0.17-4.84)	**1.66 (1.07-2.58)**

Significant associations are shown **in bold.**

NIHSS: National Institutes of Health Stroke Scale; ASPECTS: Alberta Stroke Program Early CT Score; NWU: net water uptake; eTICI: expanded Thrombolysis in Cerebral Infarction

Supplementary Table S5. Associations between variables and (the cubic root of) lesion growth, stratified by pCore volume.

**Variable**	**ß-Coefficient (95% CI)**	
	**Core ≥50 mL**	**Core <50 mL**

Age (years)	-0.05 (-0.12-0.01)	-0.01 (-0.03-0.01)
NIHSS	-0.03 (-0.09-0.15)	0.001 (-0.04-0.04)
ASPECTS	**-0.90 (-1.22-[-0.57])**	**-0.31 (-0.45-[-0.16])**
NWU (%)	**0.21 (0.06-0.35)**	**0.05 (0.01-0.08)**
Time admission to recanalization (mins)	0.003 (-0.002-0.009)	0.002 (-0.001-0.005)
Successful reperfusion (eTICI 2c/3)	**-1.91 (-3.48-[-0.35])**	**-0.72 (-1.27-[-0.17])**

NIHSS: National Institutes of Health Stroke Scale; ASPECTS: Alberta Stroke Program Early CT Score; NWU: net water uptake; eTICI: expanded Thrombolysis in Cerebral Infarction

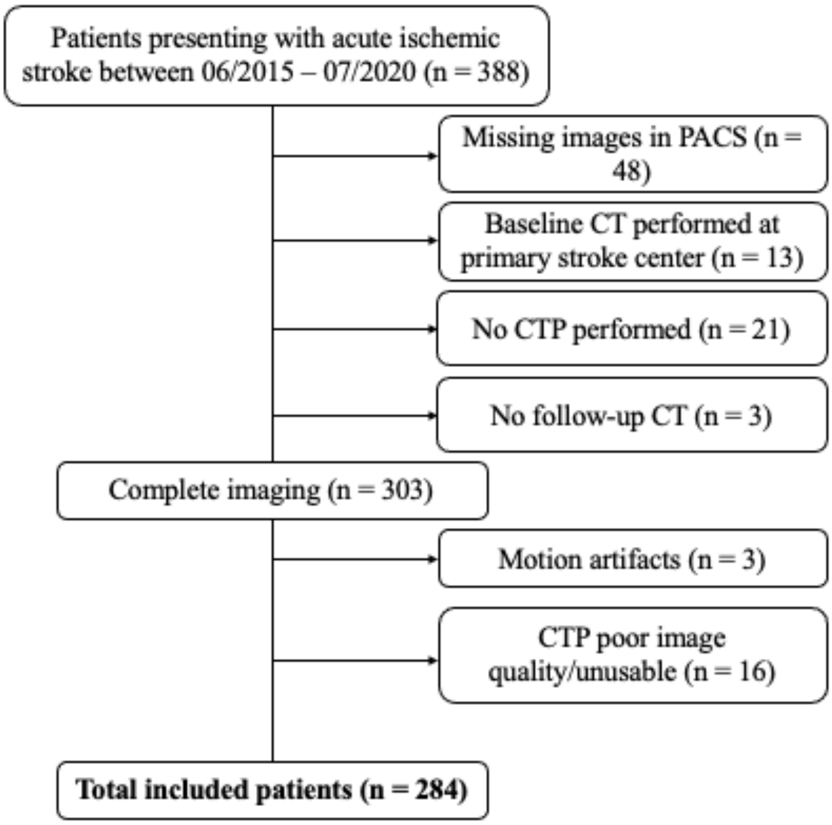

Supplementary Figure S1. Study inclusion-/exclusion flowchart. The majority of patients were excluded due to missing imaging information.
